# Data related to the life cycle assessment of 44 artisanally produced french protected designation of origin (PDO) cheeses

**DOI:** 10.1016/j.dib.2022.108403

**Published:** 2022-06-22

**Authors:** Adeline Cortesi, Laure Dijoux, Gwenola Yannou-Le Bris, Caroline Pénicaud

**Affiliations:** Université Paris-Saclay, INRAE, AgroParisTech, UMR SayFood, Palaiseau 91120, France

**Keywords:** Life cycle assessment (LCA), Food industry

## Abstract

Food production is associated with significant environmental impacts. To reduce these, it is first essential to better understand the environmental implications of production processes, and how these can vary among different products. Historically, most studies in this area have focused on comparing environmental impacts among different food categories, but here, we chose to investigate differences at a finer scale, among products belonging to the same category. Cheese was selected as our study model because it is one of the most popular types of food products around the world but is associated with significant environmental impacts. Specifically, we used Life Cycle Assessment (LCA) to evaluate the environmental impacts of 44 French cheeses that benefit from Protected Designation of Origin (PDO) certification. Our assessments took into account all steps from milk production to the end of the ripening period and the functional unit used was 1 kg of fully ripened cheese. Inventories for milk production were obtained from AGRIBALYSE 3.0. Data on cheeses production stage were collected using PDO specifications, technical datasheets, scientific literature, and three expert opinions. Background inventories (electricity mix, gas production, materials, transport, water, cleaning products) were obtained from the Ecoinvent 3.6 database. Simapro software was used to conduct all LCAs, using the characterization method "EF 3.0 Method (adapted) V1.00 / EF 3.0 normalisation and weighting set". Our final dataset contains details on products, life cycle inventories (LCI), and LCIA results. These data could be useful for cheesemakers seeking to improve the environmental performance of their products.

## Specifications Table

**Table:** 

Subject	Environmental Engineering
Specific subject area	Environmental assessment in the food industry
Type of data	Table
How data were acquired	Data used in the inventory were obtained from scientific and technical literature, product specifications, and expert opinion.The method “EF 3.0 Method (adapted) V1.00 / EF 3.0 normalization and weighting set” was performed using SimaPro software. The databases used were AGRIBALYSE 3.0 and Ecoinvent 3.6.
Data format	RawAnalysed
Parameters for data collection	44 French PDO cheeses
Description of data collection	PDO specifications were used to identify all aspects of cheese production that are subject to legal definition (milk origin, ripening time, storage time, materials, and authorized transport length).Values of energy consumption related to milk pumping and milk processing steps were estimated based on equipment datasheets. For the base scenario, the energy consumption of the ripening step was estimated using data on two experimental ripening rooms (power requirements of equipment, number of ripened cheeses, and cheese weight). The electrical consumption was then estimated as the average estimated electrical consumption of this two ripening rooms. For the alternative scenario, measured data from the literature were used [1]. Water consumption was also estimated based on the scientific literature [2]. These data were complemented by expert opinions when necessary.
Data source location	Inventory data from France were selected in the Agribalyse 3.0 database for agricultural products. Data from France were selected in the Ecoinvent 3.6 database when possible and complemented by European and global data when necessary for other flows. All of the inventory and calculated data used in this study are stored in the INRAE research centre in Thiverval-Grignon (FR).
Data accessibility	Repository name: Data INRAE Direct URL to data: https://doi.org/10.15454/JQLIOX

## Value of the Data

• This article presents a unique set of LCI and LCIA data for 44 French PDO cheeses produced at artisanal scale.
• These data represent an examination of the similarities and differences among numerous closely related food products, which could be useful for future LCAs or research studies.
• These data can also be used by cheesemakers who wish to have better knowledge of the environmental impacts of their products and production systems in order to reduce them.

## 1 Data Description

All of the inventory data used for LCA of the 44 French PDO cheeses (analyzed from milk production to the end of ripening) and the LCIA results are available in the associated dataset.

The dataset contains several files:

1. data_PDOcheeses_characterization: data on the cheese technology, milk type (cow, goat, sheep), and ripening time of each cheese studied.
2. data_PDOcheeses_coproducts: mass of the co-products (cream and whey) estimated associated with the production of 1 kg of each cheese.
3. data_PDOcheeses_allocation: allocation factors estimated and used for the steps before skimming, for the steps from skimming to draining, and for the steps after draining for each cheese.
4. data_PDOcheeses_LCI: inventory data for all steps of the production of each cheese for two ripening scenarios, calculated for a FU of 1 kg of cheese at the end of the ripening step.
5. data_PDOcheeses_LCIA: all LCIA results calculated using the characterization method “EF 3.0 Method (adapted) V1.00 / EF 3.0 normalisation and weighting set”.
6. data_PDOcheeses_specifications: links to the PDO specifications of all cheeses.
7. data_PDOcheeses_datasheets: links to datasheets used for LCI related to equipment used for milk pumping and milk processing.
8. data_PDOcheeses_distances: maximal distances allowed to transport milk and/or cheeses to be ripened, estimated on google maps according to the geographical area authorized in specifications.

## 2 Experimental Design, Materials and Methods

For this study, we followed the steps of the LCA methodology as described in [3].

### 2.1 Goal

The goal of the study was to assess the environmental impacts of 44 French PDO cheeses using attributional LCAs in order to compare them, discover major hotspots and identify potential means of improving environmental performance.

### 2.2 System Definition

**Fig. 1:**
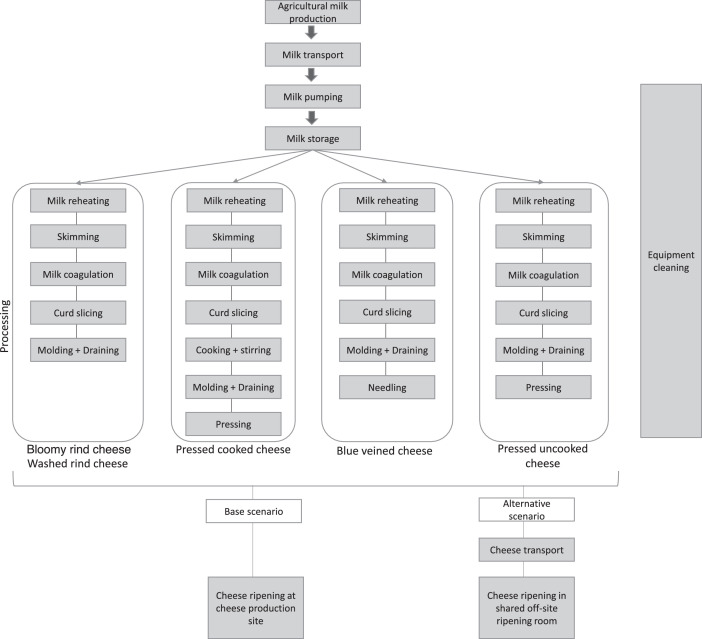
Steps of cheese production considered in the study.

The system boundary encompassed all steps going from the agricultural production of milk to the creation of the final ripened cheese product. Two different scenarios were studied. The first (base) scenario included milk production, milk transport, milk pumping, milk storage, milk processing, and cheese ripening in a small ripening room, as well as equipment cleaning. The second scenario (alternative scenario) differed from the first in that it included cheese transport to an off-site ripening room, where cheeses were ripened in a shared dedicated space (Fig. 1). The flows that were considered are milk, cream, whey, cleaning products, water, energy consumption, and equipment materials. The different inputs and outputs included in the data (e.g. inputs and emissions for electricity production, inputs and emissions linked to transport, agricultural crop production for animal feeding) were also encompassed in the system boundary. The excluded activities were all activities occurring after the ripening step such as retailing or consumption. Dry basis allocations were used to allocate the environmental impacts between cheeses and cheeses co-products.

### 2.3 Functional Unit

The functional unit was 1 kg of cheese at the end of the ripening step. This functional unit allowed us to compare the different cheeses independently of their final weight and size.

### 2.4 Inventory and Data Collection

The inventory data were mainly collected from the cheese specifications which are regulatory documents containing all the requirements the cheese need to respect in order to be allowed to be certified as a PDO. Therefore, several information such as types of milk used, the geographical areas of production, production conditions, and authorized or prohibited utensils and equipment are written there and were used for LCI. Inventory data that were not available in the specifications were obtained by interviewing three cheeses experts from the academic area in the cheese sector (acknowledged in the Acknowledgments section) and by literature research.

#### 2.4.1 Milk production

The amount of milk necessary to produce 1 kg of cheese was estimated thanks to (i) for most cheeses, the knowledge of three cheeses experts from the academic area (acknowledged in the Acknowledgments section), (ii) completed by data found on cheesemakers websites when needed. For cow milk, highland and lowland cows were considered separately. All the data are presented in the associated dataset (data_PDOcheeses_LCI). Inventory data from AGRIBALYSE 3.0 database were used for this step. Therefore, all inputs and outputs included in the background data were included in the calculation (e.g. water used for the production of animal feed, energy needed for harvesting, emissions linked to animals breeding…).

#### 2.4.2 Milk Transport

For each cheese, the maximum distance permitted by the specifications was used. All the distances used for the calculations are presented in the associated dataset (data_PDOcheeses_distances). Transportation was assumed to be land-based and refrigerated; the data used to model the refrigerated truck is available in data_PDOcheeses_LCI.

#### 2.4.3 Milk Pumping

This step corresponds to the transfer of milk from the refrigerated truck to the storage tank. We assumed that the same pump, one commonly used in dairy processes, was used for the milk transfer of all studied cheeses. The power used by the pump during the transfer was estimated from technical datasheets and electrical consumption was calculated following Eq. (1).

(1) Electricalconsumption(kWh)=Power(W)×Usetime(h)

The use time was the time needed to pump the milk amount needed calculated following Eq. (2).

(2) $$Use\;time\;\left(h\right)=\;\frac{Flow\;rate\;\left(L/h\right)}{Milk\;to\;pump\;\left(L\right)}$$

The flow rate was obtained from the technical datasheet and the amount of milk necessary to produce 1 kg of cheese was obtained as described in Section 2.4.1. The milk densities used to convert L into kg were 1.032 kg/L for cow milk, 1.023 kg/L for goat milk and 1.036 kg/L for sheep milk.

#### 2.4.4 Milk Storage

The duration of the milk storage step varied among the different cheeses, but for each cheese, we used the maximum storage time allowed in the specifications. For cheeses whose specifications did not mention storage times, estimates were made based on similar cheeses. The flows taken into account for this step were the electrical consumption linked to refrigeration and the materials of the vats. The electrical consumption was estimated according to Eq. (1) from the power needs of a refrigerated storage tank (found in a technical datasheet) and the use time being the longest storage time allowed in the specifications of each cheese.

For each cheese, the mass of material attributable to the storage step was calculated as shown in Eq. (3).

(3) $$tank\;material\;mass\;\left(kg\right)=tank\;mass\;\left(kg\right)*\frac{time\;of\;use\;\left(h\right)}{lifetime\;\left(h\right)}*\frac{milk\;\left(kg\right)}{total\;milk\;\left(kg\right)}$$

(Milk (kg): Mass of milk necessary to produce 1 kg of the cheese under consideration; Total milk (kg): Mass of the total milk capacity of the tank)

#### 2.4.5 Milk Processing

##### 2.4.5.1 Skimming

Skimming is not performed for all cheeses. We used the hypothesis that skimming is done by hand: the operator simply collects the cream that accumulates at the surface. Thus, no electrical input was considered for this step.

##### 2.4.5.2 Milk Reheating

If milk is put into cold storage after reception, it must be reheated before being processed. The electricity consumption of this step was calculated as the amount necessary to reheat the milk from its storage temperature (hypothesis: 4°C) to the optimal temperature, which is specific to each cheese. This was estimated using Eq. (4).

(4) E=mCpΔT

(E: Energy; m: milk mass; Cp: Heat capacity; ΔT: the difference between 4 °C and the optimal temperature)

##### 2.4.5.3 Milk Coagulation

For milk coagulation, only the material of the vat (stainless steel, copper, or wood, depending on the specifications) was taken into account. The calculation of the material weight attributable to each kg of cheese produced was estimated using Eq. (3).

##### 2.4.5.4 Curd Slicing

Since the focus here was on artisanal cheeses, it was assumed that curd slicing was performed manually. Therefore, no flows were taken into account for this step.

##### 2.4.5.5 Cooking and Stirring

This step occurs in the production of cooked pressed cheeses. The flows included for this step were the electricity needed for heating, and were estimated according to Eq. (1) from the use time (available in cheese specification or estimated by experts acknowledged in the Acknowledgment section) and the equipment power found in technical datasheets (available in data_PDOcheeses_datasheets). Since milk stirring is performed manually for artisanal cheeses, no flows were calculated for this action.

#### 2.4.6 Molding and Draining

The molding step is mainly done by hand for the production of artisanal French PDO cheeses.

Based on our discussions with experts, we modelled a scenario in which draining occurs spontaneously during and after molding through small holes in the mold. For this reason, no flows were taken into account for this step.

#### 2.4.7 Pressing

This step is indispensable for pressed cheeses. Following the recommendations of experts, we modelled a process of manual pressing, performed by hand with a traditional press. No flows were therefore taken into account for this step.

#### 2.4.8 Needling

Piercing is necessary to produce blue-veined cheese. This process consists of inserting needles into the cheese to form grooves where mold can develop. The assumption was made that piercing was done manually for all blue-veined cheese studied, and therefore no flows were taken into account for this step.

#### 2.4.9 Cheese Transport (for Alternative Scenario)

For the alternative scenario, we included a transportation step in which cheeses were transported from the cheesemaking facility to an off-site ripening room. As with milk transport, we assumed the longest distance allowed in the specifications for each cheese, as well as a land-based and refrigerated transport. All the distances are available in data_PDOcheeses_distances.

#### 2.4.10 Cheese Ripening

The first scenario is one in which cheeses are produced and ripened in the same location. For this scenario the average daily electrical consumption of two experimental artisanal ripening rooms (respectively 16 m² and 12.5 m^3^) were used to estimate the inventory data. The electrical consumption was estimated using Eq. (1).

For the alternative scenario in which the cheese is transported to an off-site facility and then ripened in a large shared room, an industrial ripening room of 340 m^3^ was used [1]. The electrical consumption used for this scenario was the average of three measurements of daily electricity consumption with continuous ventilation, per kg of cheese. For both scenarios the estimated energy consumption included the equipment needed to maintain the temperature in the ripening room.

Shelving/support materials (steel or wood, depending on the cheese) were also included, using a time allocation.

#### 2.4.11 Equipment Cleaning

The amount of water and detergent required to clean the various items of equipment used during cheese production was estimated using the "Reference Document on Best Available Techniques" [2].

#### 2.4.12 Co-Products

Cheese production yields cream and whey as co-products. Cream is only produced for cheeses that are made using a skimming step, while whey is produced for all cheeses, mainly during the draining and pressing step. The masses of cream and whey produced were estimated for each cheese as follows.

##### 2.4.12.1 Cheeses Produced Without a Skimming Step

The production of cheeses without a skimming step leads to the generation of only one co-product: whey. The mass of whey produced can be calculated as shown in Eq. (5).

(5) $$Whey\;mass=milk\;mass*\left(1-\%Cheese\;yields\right)*\left(1-\frac{\%\;whey\;loss}{100}\right)$$

The cheese yields were estimated using [4], and the whey losses were estimated by experts.

##### 2.4.12.2 Cheeses Produced With a Skimming Step

To estimate the mass of cream produced, Eq. (6) was used. We hypothesized that the cream produced was 40% fat.

(6) $$\begin{array}{ccc} & & Mass\;of\;cream\;produced\\ & & \;=\;\frac{fatty\;matter\;mass\;in\;initial\;milk-\;fatty\;matter\;mass\;in\;milk\;after\;skimming}{0.4} \end{array}$$

In order to determine the mass of whey produced during the draining phase, the milk mass after skimming must first be determined using Eq. (7).

(7) Milkmassafterskimming=Initialmilkmass−Massofcreamproduced

Then, Eq. (5) can be used with the milk mass remaining after skimming to estimate the mass of whey produced.

##### 2.4.12.3 Allocations

Several allocations were used in the calculations in the study.

###### 2.4.12.4 Time Allocations

Time allocations were used for equipment materials. The assumption was made that all metal and wood materials last for 30 years. The allocation factor used is presented in Eq. (8).

(8) $$Alocation\;factor=\;\frac{Use\;duration\;\left(hours\right)}{Life\;time\;\left(hours\right)}$$

###### 2.4.12.5 Dry Basis Allocation

A dry basis allocation (as recommended by PEF methodology [5]) was used to identify the impacts attributable to the cheese and those associated with the generated co-products. The equation used is presented as Eq. (9).

(9) $$AFi=\;\frac{DMi*Qi}{\sum_{i=n}^{n}\left(DMi*Qi\right)}$$

(*AF*_i_ : Allocation factor; *DM*i : Dry matter content (g/kg); *Q*i : Mass of the product i (kg))

In this way, a dry basis allocation factor was calculated for cream and whey and used as follows:

- For processing involving skimming:
  ○ Steps before skimming were allocated to cheese, cream, and whey.
  ○ Steps between skimming and draining were assigned to cheese and whey.
  ○ Steps after draining were attributed to cheese only.
- For processing without skimming:
  ○ Steps occurring before draining were allocated to cheese and whey
  ○ Steps occurring after draining were allocated to cheese only.

The dry basis allocation factors calculated for all 44 cheeses are presented in the associated dataset.

### 2.5 Characterization

The characterization method "EF 3.0 Method (adapted) V1.00 / EF 3.0 normalization and weighting set” [6] was used in SimaPro (9.1.1.7) software to calculate the LCIA of the cheeses.

## Ethics Statement

This work did not involve human subjects or laboratory animals, and therefore did not encounter any ethical issues.

## CRediT authorship contribution statement

**Adeline Cortesi:** Methodology, Formal analysis, Investigation, Writing – original draft. **Laure Dijoux:** Methodology, Formal analysis, Investigation, Writing – review & editing. **Gwenola Yannou-Le Bris:** Methodology, Formal analysis, Writing – review & editing. **Caroline Pénicaud:** Conceptualization, Methodology, Formal analysis, Writing – review & editing, Project administration, Funding acquisition, Supervision.

## Declaration of Competing Interest

The authors declare that they have no known competing financial interests or personal relationships which have, or could be perceived to have, influenced the work reported in this article.

## Data Availability

Dataset on the life cycle assessment of 44 artisanally produced French PDO cheeses (Original data) (Dataverse).
